# Helminth Products Potently Modulate Experimental Autoimmune Encephalomyelitis by Downregulating Neuroinflammation and Promoting a Suppressive Microenvironment

**DOI:** 10.1155/2017/8494572

**Published:** 2017-06-28

**Authors:** Alberto N. Peón, Yadira Ledesma-Soto, Jonadab E. Olguín, Marcel Bautista-Donis, Edda Sciutto, Luis I. Terrazas

**Affiliations:** ^1^Unidad de Biomedicina, Facultad de Estudios Superiores Iztacala (FES-I), Universidad Nacional Autónoma de Mexico (UNAM), MEX, Mexico; ^2^Laboratorio Nacional en Salud, FES-I, UNAM, MEX, Mexico; ^3^Departamento de Inmunología, Instituto de Investigaciones Biomédicas, UNAM, Mexico City, Mexico

## Abstract

A negative correlation between the geographical distribution of autoimmune diseases and helminth infections has been largely associated in the last few years with a possible role for such type of parasites in the regulation of inflammatory diseases, suggesting new pathways for drug development. However, few helminth-derived immunomodulators have been tested in experimental autoimmune encephalomyelitis (EAE), an animal model of the human disease multiple sclerosis (MS). The immunomodulatory activities of *Taenia crassiceps* excreted/secreted products (TcES) that may suppress EAE development were sought for. Interestingly, it was discovered that TcES was able to suppress EAE development with more potency than dexamethasone; moreover, TcES treatment was still effective even when inoculated at later stages after the onset of EAE. Importantly, the TcES treatment was able to induce a range of Th2-type cytokines, while suppressing Th1 and Th17 responses. Both the polyclonal and the antigen-specific proliferative responses of lymphocytes were also inhibited in EAE-ill mice receiving TcES in association with a potent recruitment of suppressor cell populations. Peritoneal inoculation of TcES was able to direct the normal inflammatory cell traffic to the site of injection, thus modulating CNS infiltration, which may work along with Th2 immune polarization and lymphocyte activation impairment to downregulate EAE development.

## 1. Introduction

MS is an autoimmune disease in which T-cells react against neuroantigens typically present on the neuron myelin sheath; the main pathophysiological mechanism of this disease is centered on an inflammation-dependent demyelination that results in heterogeneous neurological disturbances, including a flaccid-paralysis, blurred vision, impaired coordination, auditory dysfunction, urinary incontinence, pain, tingling, and muscle spasms [1]. MS highly depends on a complex interplay between T-cells and myeloid-derived cells (MDCs), such as monocytes and neutrophils. The experimental evidence [2, 3] suggests that myelin-specific T-cells may be primed in the periphery by such cells to gain access to the CNS, where they may be reactivated by local antigen-presenting cells (APCs) by self-antigen presentation in order to orchestrate the inflammatory response that induces the neurodegenerative process [4]. Conversely, activated lymphocytes produce both Th1 and Th17-type cytokines, which activate and recruit inflammatory monocytes and neutrophils, respectively [5]. Once in the CNS, both of these MDCs secrete important amounts of short-lived free radicals that degrade myelin to generate the main symptomatology [6], in such a way that the whole disease seems rather unlikely without the parenchymal invasion by leukocytes [7].

Many genetic and ontogenetic studies suggest that MS only affects genetically susceptible patients, but its triggers are mainly environmental factors [8], as many microbial infections are thought to initiate the disease [1], while others have also been shown to suppress its initiation and progression, among of which helminth infections have been pointed out in the last few years [9]. In fact, in the second half of the 20th century, a rise in the prevalence of asthma and allergies was only observed in highly industrialized countries, which correlated with a decrease in bacterial and viral infections (associated with increased hygienic habits) in the same areas. This served Strachan to propose the *hygiene hypothesis* in 1989 [10], which states that in the absence of intense infections to modulate immunity towards strong Th1 responses, Th2 immunity may be overactive, thus favoring the appearance of Th2-mediated diseases like allergies and asthma. Likewise, a few years later, the increased hygiene in industrialized countries also correlated with an increase in Th1/17-mediated autoimmune diseases, but this time chronic infections that induce Th2-immune responses (like those produced by helminth parasites) were lacking. This served to hypothesize that helminths may be important environmental regulators for tolerance induction against autoimmunity, in an addendum to the original hygiene hypothesis [11].

Furthermore, it has been thoroughly described that helminths exploit several tolerance-related pathways to regulate inflammation in their own benefit, including strong Th2-biased responses, as well as the induction of many regulatory cells such as alternatively activated macrophages (AAMs), myeloid-derived suppressor cells (MDSCs), T-regulatory (Tregs), B-regulatory (Bregs), and tolerogenic dendritic cells (iDCs), [12–14] and reviewed by Peón et al. [15]. Probably, the same bundle of regulatory strategies may be the underlying cause of the hypothetical helminth-dependent dampening of immunologic disorders, which may be useful for the treatment of autoimmune diseases.

On the other hand, to facilitate the study of MS, numerous animal models of the disease have been developed, among of which EAE stand out for its vivid emulation of the clinical signs, immunological traits, and pathophysiological mechanisms observed in patients [16, 17]. Such model has been useful in the study of the interactions between helminth infections and autoimmunity. For instance, *Taenia crassiceps* [18], *Schistosoma mansoni* [12], *Trichinella spp*. [13, 14], *Fasciola hepatica* [19], and *Strongyloides venezuelensis* [20] infections have been tested for EAE regulation with different degrees of success, and they were proven to have similar mechanisms of action involved, such as the induction of AAMs, MDSCs, Th2-differentiation, and the induction of low proliferative responses in lymphocytes. Most of these studies were performed in preinfected mice and left unanswered if the disease can be modulated after its onset and whether helminth-derived products are able to supply the infection with the live parasite.

Several immunoregulatory activities of *T. crassiceps* infection have been reported by impacting different inflammatory diseases such as colitis [21], type 1 diabetes [22] and EAE [18], mainly by modulating macrophage and DC activities [7]. Therefore, in this study, we investigated whether excreted/secreted products from *T. crassiceps* cysticerci are able to modulate EAE development when administered shortly after the onset of the disease, or even at a more advanced stage and asked about the possible mechanisms underlying its effects on EAE development.

## 2. Materials and Methods

### 2.1. Mice, Parasite Infection, and TcES Production

Six- to eight-week-old female BALB/c mice were purchased from the Harlan Laboratories (México) and were maintained in a pathogen-free environment at the FES-Iztacala (UNAM) animal facility in accordance with the Institutional and National guidelines. These mice were intraperitoneally (IP) infected with 20 metacestodes of *T. crassiceps*. *T. crassiceps* metacestodes were harvested under sterile conditions from the peritoneal cavity of infected mice after 8 weeks of infection and washed four times in ice-cold phosphate-buffered saline (PBS; 0.15 M NaCl, 0.01 M sodium phosphate, and pH 7.2) prior to maintaining them in culture into a sterile PBS at 37°C for 24 h. The supernatant was recovered and centrifuged for 10 min at 1000 g to clean it, and the heavy fraction was concentrated by a new centrifugation using 50 kDa Amicon Ultra Filter (Millipore) for 30 min at 1000 g. Protease inhibitors were added to the ≥50 kDa fraction, and samples were stored at −70°C until further use.

### 2.2. EAE Induction

EAE was induced and evaluated according to the protocol of Stromnes and Goverman [16]. In brief, a 1 : 1 emulsion mixture of 125 *μ*g myelin oligodendrocyte glycoprotein (MOG35–55) (LifeTein, NJ, USA)/100 *μ*l sterile PBS and complete Freund's adjuvant (CFA) containing 5 mg of heat-killed *Mycobacterium tuberculosis* H37RA/ml (Difco Laboratories, Detroit, MI, USA) was prepared. Female C57BL/6 mice, at 10–12 weeks old, were subcutaneously injected in four points (50 *μ*l per site) at the hindquarters with the emulsion. Pertussis toxin (600 ng/200 *μ*l sterile PBS) (List Biological Laboratories Inc., CA, USA) was administered intraperitoneally (IP) on the same day of immunization and two days later. Mice were scored every other day according to Stromnes and Goverman [16] scale to assess clinical scores: 0, no sign of clinical disease; 0.5, partial tail paralysis (loss of tone on the tip of the tail); 1, tail flaccidity; 1.5, hind limb weakness; 2, paresis in one hind limb; 2.5, paresis in one hind limb and weakness in the other; 3, complete hind limb paralysis; 3.5, paresis in hind limbs and in one forelimb; 4, total limb paralysis; and 5, moribund or death. The peak of the disease (control mice reaching score 3.5) typically occurred in 14–16 days after disease onset (typically day 26 PI) and was chosen as the ideal time for mice sacrifice to collect samples, unless otherwise stated.

### 2.3. Treatment Regimes

The EAE + TcES-treated mice received an intraperitoneal injection of 250 *μ*g TcES/200 *μ*l PBS every other day, starting (day 12 ± 2) two days after EAE onset, until sacrifice, for a total of 7 injections. The EAE + BSA-treated mice received an injection of 250 *μ*g of bovine serum albumin (BSA)/200 *μ*l of sterile PBS in the same regimen and were used as a control group for irrelevant protein administration. By contrast, the EAE + TcES-L-treatment started (day 18 ± 2) six days after the normal EAE + TcES treatment onset and were given an injection every other day until sacrifice; in this way, mice received three fewer injections than normal EAE + TcES-treated mice. The EAE + TcES-W-treated mice received the same regime as the normal EAE + TcES-group, but TcES inoculation was withdrawn from day 19 ± 1 until sacrifice (day 27 ± 1), in a way that they received three fewer injections than the normal EAE + TcES-treated group. The EAE + Dex-treated mice received a 0.3 mg/kg intraperitoneal injection of pharmaceutical grade dexamethasone suspended in 200 *μ*l of sterile PBS (according to [23]), in the same regime of the normal EAE + TcES treatment. Each group had *n* = 6 for every experiment.

### 2.4. Histopathological Evaluation

For the histological evaluation of EAE, animals were euthanized at the peak of the disease. The spinal cord was removed and fixed in absolute alcohol. The tissue samples were embedded in paraffin, and 10 *μ*m sections were cut on a microtome and stained with hematoxylin and eosin for histological examination. All the sections were cut from the lumbar spine, and the infiltration was analyzed using Axiovision LE software (Oberkochen, Germany) in randomly selected slides. The data shown are expressed as the average + standard error mean (SEM) of the total infiltrated area (selected pixels) in 5 slides per group or the number of lesions per section of the same slides. The microphotographs shown are representative of each group.

### 2.5. Cell Proliferation Assays

At the peak of EAE, spleens were aseptically removed and single-cell suspensions were prepared by perfusing the spleen with 10 ml of RPMI-1640 media supplemented with 10% FBS, 100 U of penicillin/streptomycin, 2 mM glutamine, 25 mM HEPES, and 1% nonessential amino acids (all from GIBCO, USA). The spleen cells were centrifuged at 1000 g, and erythrocytes were lysed by resuspending the cells in Boyle's solution (0.17 M Tris and 0.16 M ammonium chloride). Following two washes with sterile PBS, the viable cells were counted using Trypan blue exclusion, and the splenocytes were adjusted at 3 × 10^6^ cells/ml in the same medium. The cells (100 *μ*l per well) were seeded in 96-well plates (Costar, USA) and stimulated with 10 *μ*g/ml of MOG35–55. Additionally, the splenocytes were seeded in an anti-CD3-coated plate (10 *μ*g/ml for two hours at 37°C). The proliferation was quantified after five days of incubation at 37°C and 5% CO_2_ by pulsing the cells for 24 h with 0.5 *μ*Ci [^3^H]-thymidine (Amersham Biosciences, USA). The cells were harvested on a 96-well harvester (Tomtec, Finland) then counted using a 1450 micro*β*-plate counter (Trilux, Finland). The values are presented as counts per min (CPM) from triplicate wells. Supernatants from similar cultures without [^3^H] thymidine were frozen and stored at −70°C until used for detection of cytokines.

### 2.6. ELISA Sandwich

The levels of IL-4, IL-10, IL-17A, TNF-*α*, and IFN-*γ* were quantified in blood serum and splenocyte culture supernatants at the indicated time points. ELISA kits were used according to the manufacturer's instructions (Peprotech, México or Biolegend, USA for IL-17).

### 2.7. Isolation of CNS Inflammatory Cell Infiltrates

The mice were anesthetized using a mix of ketamine/xylazine 3 : 1 to perform a transcardial perfusion with a sterile gPBS (glucose-additional PBS) containing 0.002% of glucose in 1× PBS. The mice were then sacrificed and the whole brains and spinal cords were obtained and washed with this solution. The nervous tissue was disaggregated and centrifuged at 400 g for 10 min at 23°C to concentrate the cellular fraction, which was resuspended and digested in 5 ml of PBS containing 500 U of DNAse and 15 U of collagenase for 1 h at 37°C. The digested CNS tissue was washed in 45 ml of gPBS solution and resuspended in 4 ml of 30% percoll, which was added on top of two more gradients containing 37% (medium layer) and 70% (bottom layer) percoll to be centrifuged at 500*g* for 20 min at 23°C. The medium percoll layer containing the inflammatory infiltrate was obtained and washed with 45 ml of gPBS, and the resulting pellet was then prepared for the analysis of the surface marker expression using flow cytometry.

### 2.8. Cell Isolation from Peritoneal Cavity, Blood, and Lymphoid Organs

Peritoneal exudate cells were extracted by washing the peritoneal cavity of mice with 15 ml of sterile Hank's solution, which was recovered after a soft massage of the ventral area to be concentrated and cleaned by two successive centrifugations. Total splenocytes were extracted by spleen perfusion with 10 ml of the same solution using 25G needles in a Petri dish; cells were deprived from erythrocytes by resuspending the samples in Boyle's solution (0.17 M Tris and 0.16 ammonium chloride) and afterward cleaned with sterile Hank's solution. Blood was extracted from the base of the tail, and 800 *μ*l of the samples were incubated in EDTA-coated tubes (Becton Dickinson, USA) in gentle rocking motion for 20 min; afterward, erythrocytes were lysed as described and cells were washed in preparation for staining. All samples were collected and treated in aseptic conditions.

### 2.9. Analysis of Surface Markers

The surface lineage and activation-type markers were analyzed using multicolor flow cytometry. Peritoneal exudate cells (PECs), splenocytes, cervical lymph node (CLNs) cells, blood, and CNS-infiltrating cells were extracted, washed, and suspended in FACS buffer (PBS/FBS 0.5%/0.05% NaN_3_). Fc receptors were blocked with 1 *μ*g/ml of CD16/32 for 30 min at 4°C. Cells were washed and stained with antibodies against CD11c, Ly6C, Ly6G, F4/80, PD-L1, PD-L2, CD4, and CD8 (all antibodies from Biolegend) and analyzed on a FACSCalibur or FACSAria fusion flow cytometer using Cell Quest software (Beckton Dickinson) or Flowing Software 2 (Perttu Terho, Finland).

### 2.10. Intracellular Cytokine Staining

Splenocytes free of red blood cells were washed with complete RPMI 1640 media and adjusted 3 × 10^6^ cells/ml. Two ml per well of the cell suspension were incubated in a 24-well plate, stimulated with 5 ng/ml of phorbol 12-myristate 13-acetate (PMA) (Sigma) plus 500 ng/ml of ionomycin (Sigma), and 10 *μ*g/ml of brefeldin A (Biolegend) at 37°C, 5% CO_2_ for 4 hours. Cells were recovered into FACS tubes containing 1 ml of ice-cold FACS buffer and centrifuged at 1500 rpm for 5 min. After staining the cell surface with the indicated lineage markers, cells were washed and fixed, using 2% paraformaldehyde in PBS for 10 min and then washed with intracellular staining buffer (PBS, 1% FCS, and 0.025% saponin) and resuspended in 100 *μ*l of the same buffer containing the indicated mAbs (all from Becton Dickinson, México) for cytokines for 10 min. The cells were again washed and resuspended in 500 *μ*l of FACS buffer and analyzed on a FACSAria Fusion cytometer (Becton Dickinson, USA).

### 2.11. Statistical Analysis

All data were presented as mean ± SEM. The significance of the differences between the experimental groups was measured using a two-tailed Student's *t*-test. The significance in EAE progression between groups was measured by two-tailed ANOVA of both the cumulative disease index (CDI) and the area under the curve (AUC), independently. CDI is the sum of all grades evaluated per mice in each group. The differences were considered significant by the following criteria: ^∗∗∗^*P* < 0.001, ^∗∗^*P* < 0.01, and ^∗^*P* < 0.05.

## 3. Results

### 3.1. Repeated Administration of TcES (250 *μ*g Dose) Induced a Th2-Type Response In Vivo

**Figure 1:**
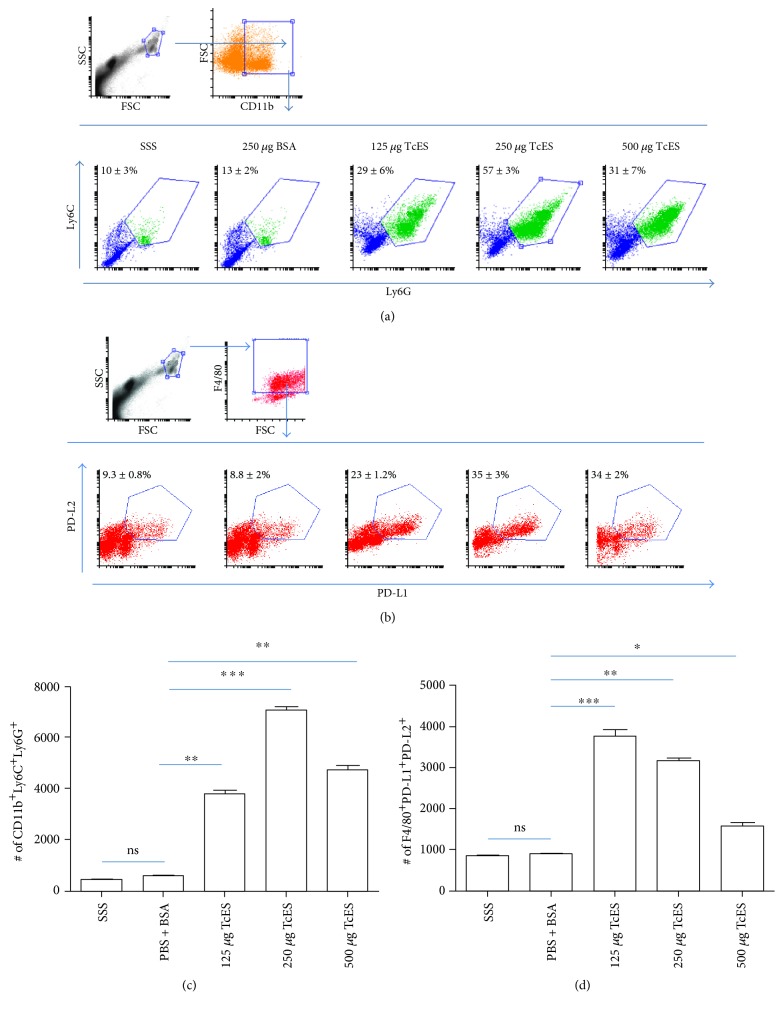
A 250 μg dose of TcES best recruits MDSCs and AAMs. Frequencies (a) and numbers (c) of MDSCs recruited to the peritoneal cavity of mice treated with different doses of TcES or control treatments (SSS, BSA). Frequencies (b) and numbers (d) of F4/80^+^ PD-L1^+^ PD-L2^+^ AAMs. Cell numbers were calculated from a gate of 10000 total cells. The data shown are representative of 2 independent experiments. Means and SEM were calculated for groups of *n* = 4. The statistical significance between groups was determined by two-tailed Student's *t*-test, ^∗∗∗^*P* < 0.001, ^∗∗^*P* < 0.01, and ^∗^*P* < 0.05. SSS, sterile saline solution; BSA, bovine serum albumin; TcES, *Taenia crassiceps* secreted/excreted; MDSC, myeloid-derived suppressor cells; AAMs, alternatively activated macrophages.

In a previous work, it was found that an 8-week-long infection of C57BL/6 mice with *T. crassiceps* induced a suppressive milieu rich in alternatively activated macrophages (AAMs), myeloid-derived suppressor cells (MDSCs), and IL-10, which was able to regulate the EAE development [18]. The present work investigates if TcES was able to emulate the immunoregulatory effects of the *T. crassiceps* infection and its impact on the EAE development. For this, *T. crassiceps* metacestodes were cultivated in sterile PBS and we concentrated the TcES products ≥50 kDa fraction. Two batches were made to perform all the experiments, and their molecular integrity and consistency were evident by comparison between batches in an SDS-PAGE gel (Figure S1 in Supplementary Material available online at https://doi.org/10.1155/2017/8494572). Three different amounts of TcES (125, 250, and 500 *μ*g) were tested in healthy C57BL/6 female mice in the same inoculation regime to find the optimal dose of TcES to induce a regulatory environment similar to the one induced by the infection. The inoculation regime consisted of one IP injection every other day for 16 days, and the results were compared against two control treatments: healthy mice receiving sterile saline solution (SSS) as a vehicle control and another group that received 250 *μ*g of bovine serum albumin (BSA) in SSS as an irrelevant protein control. Blood sera were extracted from the base of the tail from all mice at day 1 (prior to treatment), day 8, and day 16 after the respective inoculation. The levels of IL-4, IL-10, and IFN-ɣ were measured to test for any changes, and only the 250 and the 500 *μ*g treatments were able to induce increased levels of IL-10 that were detectable from the midpoint to the endpoint of the treatment, whereas no dose was able to induce any detectable change in IL-4 secretion, but the 500 *μ*g dose of TcES elevated the levels of the proinflammatory cytokine IFN-ɣ at the endpoint (Figure S2). Accordingly, the 250 *μ*g doses of the TcES induced the most potent peritoneal recruitment of MDSCs (defined as CD11b^+^/Ly6C^+^/Ly6G^+^ cells) (Figures 1(a) and 1(c)) and AAMs (defined as F4/80^+^/PD-L1^+^/PD-L2^+^ and F4/80^+^/MMR^+^/IL-4R*α*^+^ cells) (Figures 1(b) and 1(d) and Figure S3). Therefore, the 250 *μ*g doses of the TcES were selected for its test in the EAE-induced mice, and the PBS + BSA treatment was chosen for the control group.

### 3.2. TcES Treatment Foster Protection against EAE Development

**Figure 2:**
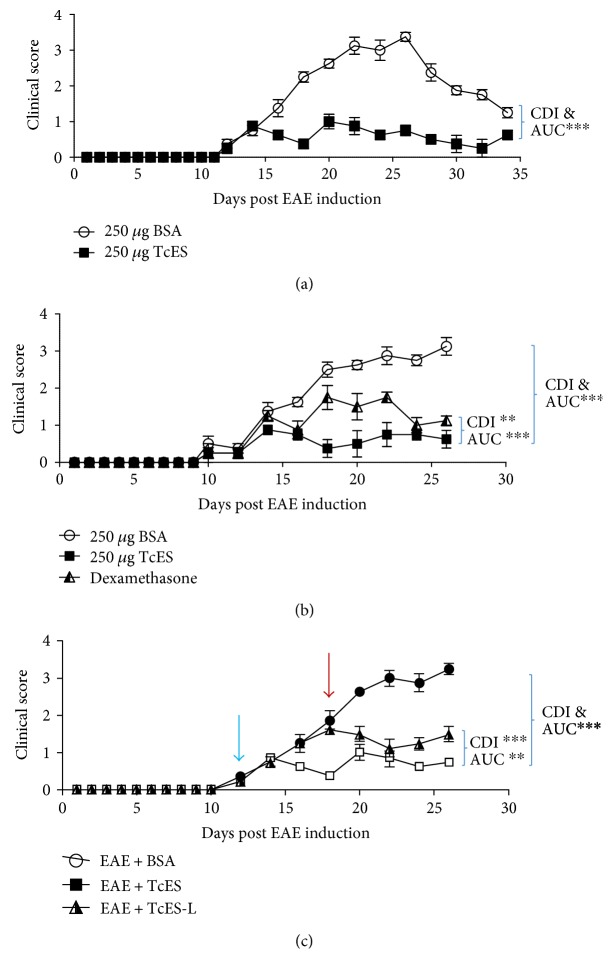
TcES foster protection against EAE development with more potency than dexamethasone. The TcES treatment significantly reduced EAE clinical score after a whole clinical course (a). The TcES treatment modulates EAE better than a high dose of dexamethasone (0.3 mg/kg every other day); treatments started 2 days after disease onset (day 10) and continued until sacrifice at the peak of disease (day 26 PI) (b). The TcES modulated disease even when inoculated at a later point in time (day 18 PI, red arrow; normal TcES treatment started at day 12 PI, blue arrow) upon continued treatment until the peak of disease (day 26 PI) (c). Data shown are representative of 2–4 independent experiments. Means and SEM were calculated for a group of *n* = 6 mice. The difference between AUC and CDI were calculated by two-tailed ANOVA test and described by the following criteria: ^∗∗∗^*P* < 0.001 and ^∗∗^*P* < 0.01.

EAE was induced in 10 to 12 week-old female C57BL/6 mice by inoculating them with an MOG35–55/CFA emulsion. The disease onset occurred at day 12 ± 2 postinduction (PI) and progressed to peak at day 26 PI. Afterwards, disease intensity started to decline, and by day 36, mice fully entered in remission. The TcES treatment with the previously defined regime (250 *μ*g TcES/mouse every other day for 16 days) was able to suppress the evolution of the entire pathological phase when administered since two days after disease onset until the end of the course of pathology (Figure 2(a)), in comparison with EAE + BSA mice. Despite mice were not completely cured, the progression of the symptomatology was significantly inhibited following the TcES treatment as evidenced by a remarkable reduction in the average grade, maximum grade, AUC, and CDI (Table 1).

To further test the regulatory potency of TcES over EAE, the treatment's modulatory ability against that of a high dose of dexamethasone (0.3 mg/kg) was compared, according to Donia et al. [23]. Both treatments started at the onset day of EAE and endured until sacrifice, and mice were treated and evaluated every other day. As expected, dexamethasone was able to potently regulate the signs of EAE, but interestingly, TcES was able to induce a more significant suppression of the pathology throughout the entire period of the treatment (Figure 2(b)). In fact, the TcES treatment was twice as potent at regulating the average grade of the disease and strongly modulated the maximum grade, AUC, and CDI (Table 2), as compared to the dexamethasone treatment.

In most cases of MS, patients are treated with anti-MS drugs at the beginning of their relapses, but misdiagnosed cases of relapsing-remitting MS or in the progressive phase of the disease, MS may be treated in more advanced stages. To investigate whether TcES was able to modulate advanced EAE, a new group of mice that started to receive the treatment at a later time-point (EAE + TcES-L) than the normal EAE + TcES group (normal TcES treatment begun at day 12 PI whereas the late TcES treatment begun at day 18 PI) was added and compared to the disease evolution of both. The CDI, AUC, maximum grade, and an average grade of the EAE + TcES-L mice were lower than those observed in the control group, suggesting that the treatment with TcES was still able to arrest the EAE development even when inoculated at a later stage of the disease (Figure 2(c)). Remarkably, the late CDI (calculated parting from the late treatment onset to sacrifice) was also lower than that of the untreated group (Table 3), even when disease seemed to progress as quickly as in the untreated group prior to treatment onset. Nevertheless, the late treatment seems to arrest disease progression rather than reduce clinical scores (Figure 2(c)).

### 3.3. TcES Induce a Suppressive Th2-Type Environment

In order to further evaluate the in vivo immunological environment induced during the TcES treatment, the EAE-induced mice were sacrificed at the end of the treatment period and the total splenocytes were extracted and cultured with either a polyclonal (anti-CD3) or antigen-specific (MOG35–55) stimulation to test whether the TcES-treatment was able to induce changes in the cytokine milieu and in lymphocyte responsiveness.

**Figure 3:**
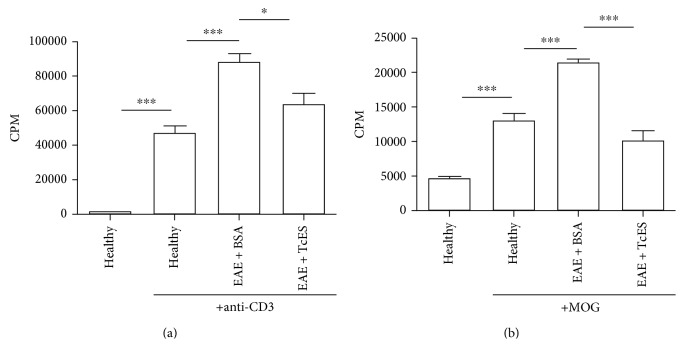
TcES induce a strong systemic suppressive environment. The TcES treatment reduces lymphocyte proliferation to polyclonal stimuli (a). The TcES treatment reduces lymphocyte proliferation to antigen-specific stimuli. The data shown are representative of 2 independent experiments where *n* = 6. Statistical significance between groups was determined by two-tailed Student's *t*-test, ^∗∗∗^*P* < 0.001 and ^∗^*P* < 0.05.

To ensure the function of our stimuli, we cultured splenocytes from normal, healthy mice (healthy) and added either an anti-CD3 antibody or MOG35–55 to the wells, and as Figures 3(a) and 3(b) depicts, the addition of the stimuli provoked the proliferation even on such cells, reflecting the existence of autoreactive lymphocytes in the case of MOG-stimulated cells and the natural ability of lymphocytes to proliferate in the case of the anti-CD3-stimulated cells. When splenocytes were cultured in the anti-CD3-coated plates and pulsed with ^3^H-thymidine, a lower level of proliferation was detected in the splenocytes that came from EAE + TcES mice than in those from EAE + BSA mice, which abundantly proliferated because of the underlying inflammatory process of the disease (Figure 3(a)). Importantly, a more conspicuous suppression of splenocytes' proliferation was detected in the EAE + TcES-treated mice when MOG35–55 was added as an antigen-specific proliferative stimulus (Figure 3(b)), suggesting that the treatment with TcES was not only able to induce a general hyporesponsive environment, but TcES was also able to greatly suppress the pathogenic MOG-specific response.

**Figure 4:**
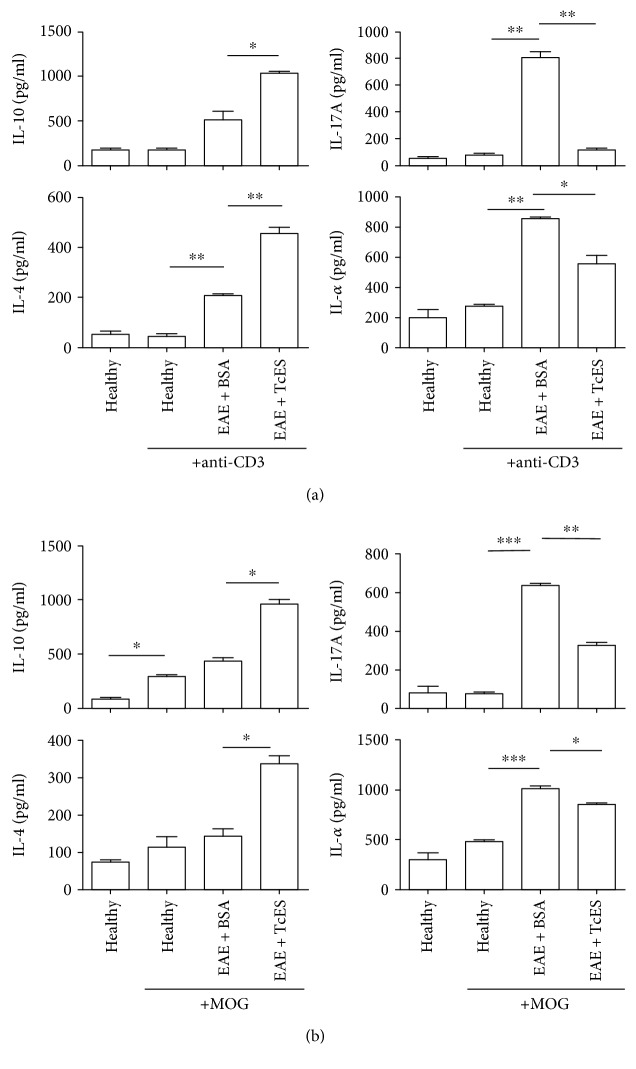
TcES induce deep changes in the systemic cytokine milieu. Splenocytes from the TcES-treated mice produce different levels of inflammatory (IL-17A, TNF-*α*) or anti-inflammatory (IL-4, IL-10) cytokines in response to polyclonal (a) or antigen-specific (b) stimuli. The data shown are representative of 2 independent experiments where *n* = 6. Statistical significance between groups was determined by two-tailed Student *t*-test, ^∗∗∗^*P* < 0.001, ^∗∗^*P* < 0.01, and ^∗^*P* < 0.05.

Furthermore, both the polyclonal and the antigen-specific stimulated cultures revealed that the splenocytes coming from the EAE + TcES mice secreted higher levels of both IL-10 and IL-4, in comparison to EAE + BSA mice (Figures 4(a) and 4(b)). Also, both TNF-*α* and IL-17A levels were dampened in the cultures of splenocytes coming from the EAE + TcES animals in comparison with those that came from the EAE + BSA mice, either measured in a polyclonal or an antigen-specific setting (Figures 4(a) and 4(b)).

### 3.4. Withdrawal of TcES Treatment Abrogates Th2 Milieu Induction and Treatment Efficacy

**Figure 5:**
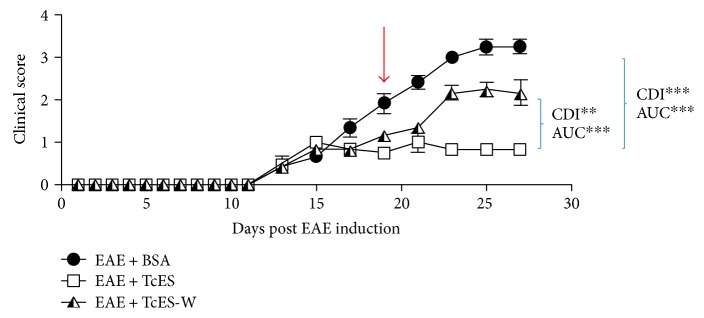
TcES withdrawal reduces treatment efficacy. All treatments started at disease onset (±day 13 PI) and continued until sacrifice (day 27 PI), but the treatment was suspended on day 19 PI (red arrow) for the EAE + TcES-W group. The data shown are representative of 2 independent experiments. Means and SEM were calculated for a group of *n* = 6 mice. The statistical significance between groups was determined by a two-way ANOVA test of the CDI and AUC, ^∗∗∗^*P* < 0.001 and ^∗∗^*P* < 0.01.

Next, a pursuit was made to determine if the found improvement in EAE development was dependent upon the constant administration of TcES. To achieve this, two EAE-ill mice groups were treated with TcES starting at the onset day, one group (EAE + TcES) received the entire treatment (one 250 *μ*g IP injection of TcES every other day, starting from disease onset day until sacrifice, for a total of eight doses) while the other group (EAE + TcES-W) started receiving the treatment at disease onset, but it was withdrawn in the middle of the treatment scheme (day 19 PI), though all mice were kept alive for comparison against EAE + TcES and EAE + BSA mice. Four days after the TcES treatment withdrawal (EAE + TcES-W), the disease clinical score started to advance in comparison with that of the TcES-treated mice, and although it never reached the grade of EAE + BSA mice, it was considerably higher than that observed in EAE + TcES group (Figure 5). Indeed, the EAE + TcES-W mice displayed lower maximum grade, CDI, and AUC than EAE + BSA mice, but higher than that of the EAE + TcES-group, and interestingly, most of the disease development occurred after treatment withdrawal, as evidenced by late CDI, which was measured from the end of the TcES treatment to the end point of the whole experiment (Table 4).

**Figure 6:**
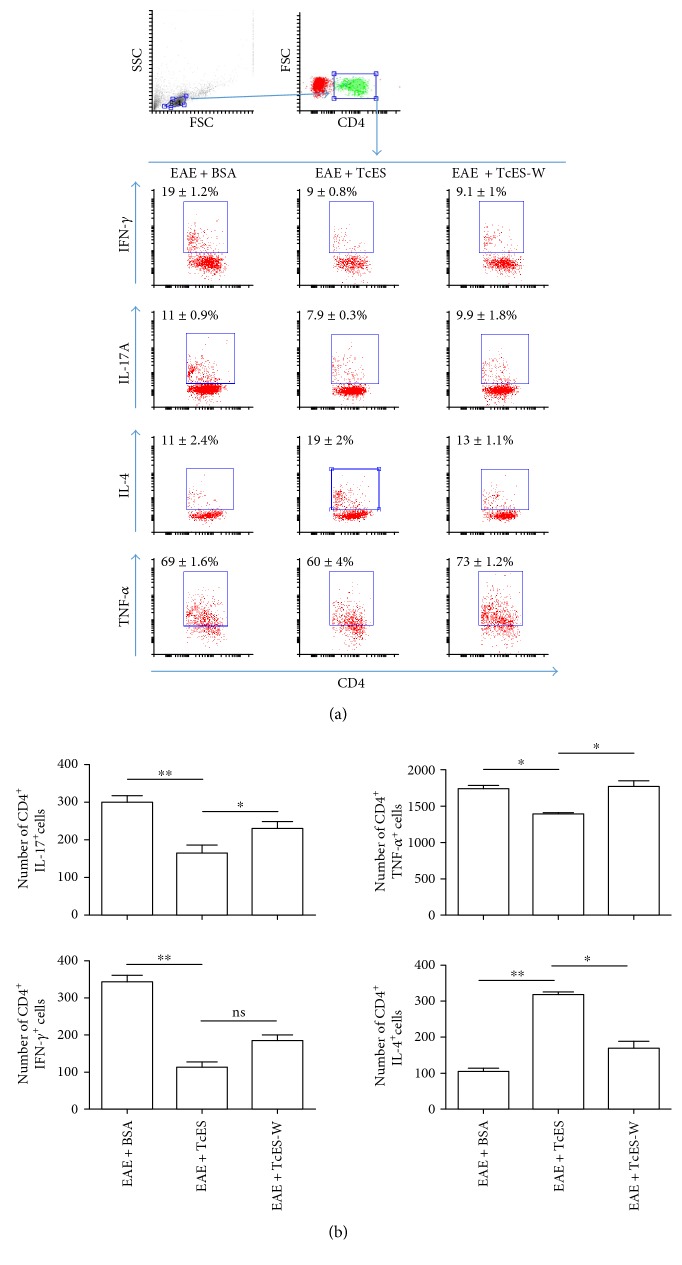
Suppressive milieu is partially reverted by TcES withdrawal. Total splenocytes were obtained from treated (EAE + TcES), control-treated (EAE + BSA), and mice that only received early treatment (EAE + TcES-W) after 16 days of receiving the mentioned treatments and were intracellularly stained to measure cytokines in the CD4^+^ lymphoid region. Frequencies (a) and numbers of CD4^+^ cytokine^+^ cells per 10000 cells gated are shown (b). All the analysis were performed from a small and nongranular gate. The data shown are representative of 2 independent experiments. Means and SEM were calculated for a group of *n* = 6 mice. The statistical significance between groups was determined by two-tailed Student's *t*-test, ^∗∗^*P* < 0.01 and ^∗^*P* < 0.05.

Moreover, while the TcES treatment increased the frequency and number of CD4^+^ IL-4^+^ cells in the spleen, the withdrawal of the treatment reduced such population almost to the same level as that found in EAE + BSA mice. The opposite happens to the CD4^+^ IL-17A^+^ and CD4^+^ TNF-*α*^+^ cells, as the constant treatment with TcES maintained these populations at lower levels, whereas TcES withdrawal allows for the increase in both populations (Figure 6).

### 3.5. Administration of TcES Directs Inflammatory Cell Infiltration to the Peritoneal Cavity Instead of to the CNS

**Figure 7:**
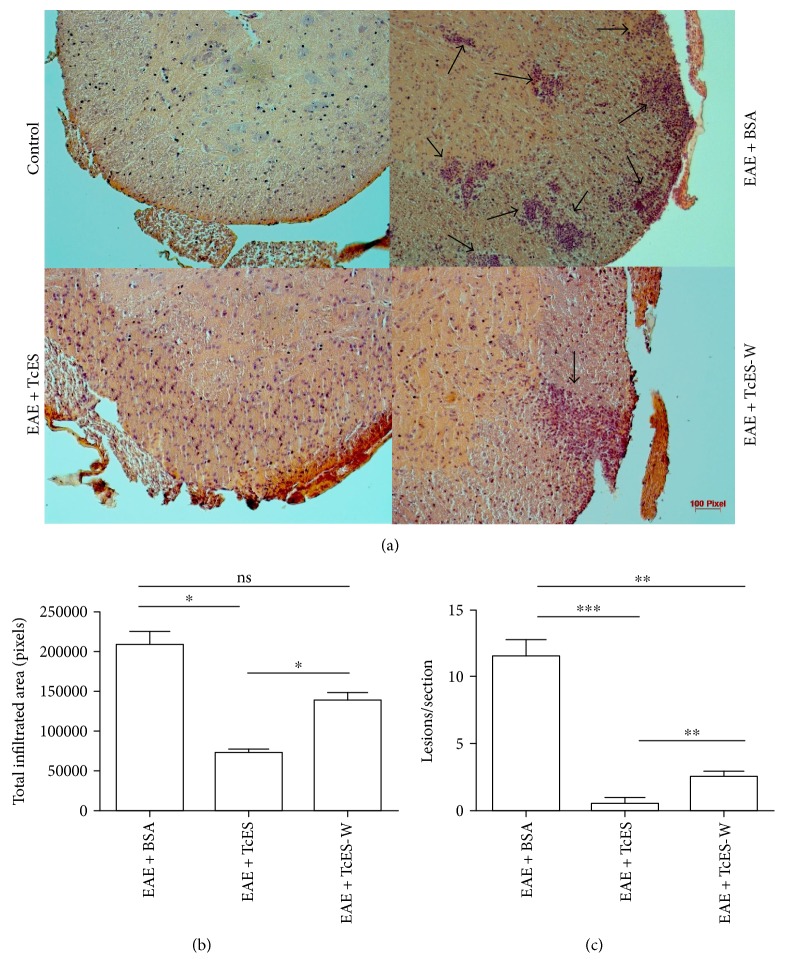
TcES treatment abrogates CNS-infiltration. At the end of the experiment, mice were sacrificed to extract the lumbar region of the spinal cord and stain it with H&E to reveal inflammatory infiltration, as pinpointed by arrows in (a). The total infiltrated area of the 5 randomly selected sections per group was calculated and plotted (b), and the number of the lesions per section (c) present on every selected slide was counted to calculate mean and SEM. The data shown are representative of 2 independent experiments. Means and SEM were calculated for a group of *n* = 5 mice. The statistical significance between groups was determined by two-tailed Student's *t*-test, ^∗∗∗^*P* < 0.001, ^∗∗^*P* < 0.01, and ^∗^*P* < 0.05. EAE + BSA, bovine serum albumin-treated EAE-disease mice; TcES, *T. crassiceps* secreted/excreted; TcES-W, TcES treatment withdrawn.

Organ-specific autoimmune diseases, such as MS/EAE, share the common feature of the parenchymal invasion by immune cells. Without the development of infiltration, no neurodegenerative processes occur in MS/EAE as most autoimmune effector and inducing/regulatory disease mechanisms are derived from infiltrated MDCs [24], such as the short-lived free radicals that mediate myelin degradation [6]. For these reasons, the pursuit was made to determine if the TcES treatment was able to downmodulate the inflammatory CNS infiltration. To study such phenomenon, the spinal cords and brains of EAE + TcES, EAE + BSA, and early-treated only (EAE + TcES-W) mice were extracted to analyze the inflammatory cell infiltration of the CNS parenchyma. The H&E stained sections of the spinal cord showed that most EAE + TcES mice lacked any inflammatory infiltrate (Figure 7(a)) and that when the parenchymal invasion of the CNS occurred, the lesions were scarce and small (Figures 7(b) and 7(c)) in sharp contrast with the EAE + BSA mice, which developed increased numbers of lesions that account for an extended infiltrated area of the total section [7]. Moreover, when the TcES-treatment was withdrawn, mice developed few but big lesions in the spinal cord (Figures 7(b) and 7(c) which correlated with an increased disease activity (Figure 5 and Table 4).

**Figure 8:**
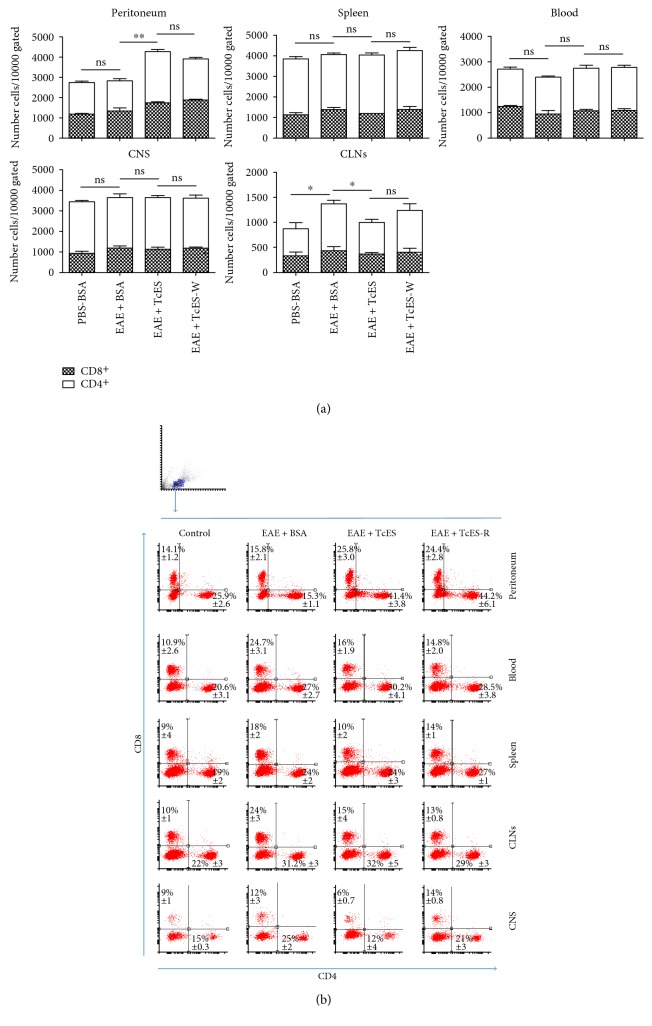
TcES treatments alter lymphoid cell migration patterns. Leukocytes were obtained from the peritoneum, spleen, CLNs, CNS, and blood and were stained to detect CD4^+^ and CD8^+^ cells. Numbers (a) and relative frequencies of either CD4^+^ or CD8^+^ cells are shown (b). Cells were gated from a small and nongranular region to plot CD4^+^ & CD8^+^ cells. Cell numbers were calculated from a gate of 10000 total cells. The data shown are representative of 2 independent experiments. Means and SEM were calculated for a group of *n* = 6 mice. The statistical significance between groups was determined by two-tailed Student's *t*-test, ^∗∗^*P* < 0.01 and ^∗^*P* < 0.05. No statistically significant differences were found in CD8^+^ cells.

**Figure 9:**
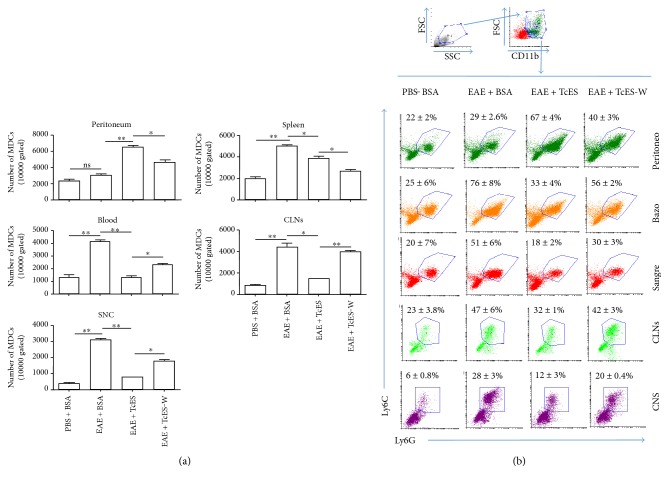
TcES treatments direct myeloid cell migration from the CNS to the peritoneal cavity. Leukocytes were obtained from the blood, spleen, CLNs, CNS, and peritoneal cavity of mice to stain CD11b^+^ Ly6C^+^ Ly6G^+^ (MDCs) cells. Numbers of cells (a) and frequencies (b) are shown. Cell numbers were calculated from a gate of 10000 total cells. Big and granular cells were gated to plot a big and CD11b^+^ gate; all data analysis was made from the latter. The data shown are representative of 2 independent experiments. Means and SEM were calculated for a group of *n* = 6 mice. Statistical significance between groups was determined by two-tailed Student's *t*-test, ^∗∗^*P* < 0.01 and ^∗^*P* < 0.05.

To further analyze the ability of TcES to inhibit CNS infiltration, the spinal cord and brains of the different groups of mice were extracted and infiltrated cells were isolated by percoll gradients to perform a flow cytometric analysis of CD4^+^ or CD8^+^ T-cells and CD11b^+^ Ly6C^+^ Ly6G^+^ (MDCs). In order to describe TcES' mechanisms of action in more detail, the same lineage markers in cells obtained from the peritoneum, spleen, blood, and cervical lymph nodes (CLNs) were also stained in such a way that we would be able to detect whether TcES treatment is able to alter the normal migration patterns of the infiltrating cells. It has been described that both the T-cells and the MDCs are concentrated in the spleen and CLNs during the priming phase of the autoimmune response [4], and then, they migrate towards the blood to the CNS, which reflects in this study's results (Figures 8(a) and 8(b) and Figures 9(a) and 9(b). Furthermore, when TcES is administrated, such cells appear to be drawn from the blood, spleen, CLNs, and CNS to be recruited into the peritoneal cavity (Figures 8(a) and 8(b), Figures 9(a) and 9(b), and Figure S4), but when TcES treatment is suspended, the peritoneal numbers of such cells decay to rise again in the blood, spleen, CLNs and specially the CNS (Figures 9(a) and 9(b)), or in the CNS alone in the case of T-cells (Figures 8(a) and 8(b)), suggesting that TcES may be able to abrogate CNS infiltration by attracting T-cells and MDCs to the peritoneal cavity, instead of letting them infiltrate the CNS, where they can induce neurodegeneration.

## 4. Discussion

MS is one of the most extended and important autoimmune diseases affecting the CNS, and EAE is the most utilized model to study new treatment options as well as new pathways for its regulation. Helminths have been largely associated with potent immunoregulatory activities and in the last decade have been used to study their potential ability to downmodulate inflammatory diseases [7]. As stated earlier, the ability of many helminth infections to modulate EAE has been tested. In such regard, all *T. crassiceps* [18], *S. mansoni* [12], *T. pseudospiralis* [14]*, T. spiralis* [13], and *F. hepatica* [19] infections have been shown to potently modulate EAE in association with a Th2-type response that is low in TNF-*α*, IFN-*γ*, and IL-17A. Also, several regulatory cell populations were detected in these trials, such as AAMs and MDSCs; all of them are able to suppress T-cell proliferation, suggesting that the regulation of EAE by a helminth infection work by the induction of such changes in the immune response. Moreover, helminths ability to inhibit CNS infiltration appear to be another immunological trait for the regulation of EAE as the infection with *S. venezuelensis* [20] was unable to modulate the disease outcome despite being able to induce some of these changes, but unlike the other helminths tested, it could not reduce CNS infiltration. Furthermore, *T. crassiceps* infection was able to downregulate the development of other inflammatory diseases such as colitis [21] and type 1 diabetes [25] in association with similar regulatory mechanisms, while also arresting the inflammatory infiltration to the target organs, thus suggesting that helminths act to inhibit inflammation in multiple ways, but the deterring of the inflammatory infiltration of the target organs seems to be of paramount importance.

A limited number of studies have been focused on the role of helminth-derived products to mimic the effect of the whole infection in the regulation of EAE [26–28]. Here, based on previous studies using the whole parasite [18], we used *Taenia*-derived factors (TcES) to evaluate their impact on EAE development. This study's main findings are that the administration of TcES modulated the EAE development with great potency, despite being inoculated shortly after the onset of disease, or when a more conspicuous or advanced pathology is happening. The potency of the treatment was even superior to that observed for dexamethasone, provided its administration starts early within the first pathological phase. Importantly, many anti-inflammatory traits are associated with TcES treatment effectiveness to include a Th2-type environment rich in IL-4 and IL-10, and the downregulation of TNF-*α*, IFN-*γ*, and IL-17A, as well as the recruitment of suppressor cell populations like MDSCs and PD-L1^+^ PD-L2^+^ AAMs. Moreover, these changes appear to induce a low proliferative response in T-cells that was even lower in MOG-stimulated cells than in those stimulated by polyclonal anti-CD3 antibodies. Additionally, the inhibition of the inflammatory infiltration of the CNS appeared to be important in the downmodulation of the pathology, as demonstrated by the histological and flow cytometry analysis of infiltrating cells in the nervous parenchyma. In line with this study's results, recently an *F. hepatica*-derived peptide was reported to potently inhibit EAE development by preventing the traffic of autoreactive cells from the periphery to the CNS [29], but interestingly, the authors were unable to find any immunological effect attributable to the inoculation of such parasite-derived peptide. In contrast, as mentioned above, the TcES treatment was able to generate more immunomodulatory effects on EAE, suggesting a more complex interplay of suppressing mechanisms for such enriched fraction.

EAE has been strongly associated with exacerbated Th1 and Th17 responses [30], as many pathogenic actions have been described for both types of responses. For instance, IL-17A downregulates the expression of the tight-junction proteins, increasing the blood-brain barrier (BBB) permeability and thus facilitate inflammatory cell infiltration into the CNS [31]. Also, IL-17A recruits and activates neutrophils into the CNS where they induce damage through ROS-dependent mechanisms [32] and promote the apoptosis of oligodendrocytes, along with high levels of TNF-*α*, thus inhibiting myelin regeneration [33]. On its own, TNF-*α* activates and recruits monocytes into the CNS to promote neurodegeneration through NO production and impair the differentiation and function of neuroprotective cell populations [34]. Thus, the regulation of both cytokines appears to be important for disease depression. Moreover, a Th2 milieu rich in IL-4 and IL-10 seems ideal for EAE dampening as it has been demonstrated that both cytokines correlate with milder disease development and lower Th1/17 responses [35]. In this regard, *T. crassiceps* and its E/S products (a group of glycoproteins with >50 KDa molecular weight) was shown to lower the activation of both murine and human DCs to several TLR ligands, mainly by inducing cRAF phosphorylation, which inhibits p38 and NFkB signaling, thus regulating the secretion of proinflammatory cytokines [36–38]. Also, TcES inhibit macrophage response to IFN-ɣ by downregulating STAT1 phosphorylation by activating SHP1 and SOCS3 [39], modulating their ability to secrete Th1-driving cytokines such as IL-12, as well as NO production, while fostering their ability to induce a Th2-type environment. Therefore, these effects at a cellular level may account for the shift to the anti-inflammatory protective response that was detected.

As EAE is mediated by T-cells, the inhibition of their proliferation and activation is another strategy of great importance for symptom alleviation. In fact, the programmed death-1/programmed death ligand (PD-1/PD-L) pathway has been linked to the regulation of EAE for its ability to downregulate the T-cell proliferation [40]. In specific, PD-L1^+^/PD-L2^+^ myeloid-derived APCs have been found to modulate EAE through lymphocyte-proliferation control [41]. Here, we found that the TcES treatment was able to induce PD-L1 and PD-L2 expression on macrophages during the EAE development, a fact that was not observed in the EAE + BSA-treated mice, suggesting a specific effect that could have had important repercussions in the induction of the low proliferative response in T-cells and the suppression of their Th1/Th17 differentiation. For such reasons, we reasoned to be possible that the recruitment of MDSCs and F4/80^+^/PD-L1^+^/PD-L2^+^ macrophages may also have a remarkable impact on the regulation of EAE.

On the other hand, it has been thoroughly described that CNS-infiltrated monocytes [42, 43] and neutrophils [6, 44] mediate demyelination through in situ secreted short-lived free radicals, and thus, their CNS invasion is critical for disease development, as it also happens with T-cells [4]. Moreover, several anti-MS drugs work through the abrogation of CNS infiltration [45], either by promoting BBB repair [46], downmodulating [47] or blocking [48] vascular cell adhesion factors, or by directing the inflammatory cells to the periphery [49, 50]. Either way, parasites and their products seem to have in common the inhibition of CNS invasion [7, 29]. In the present study, TcES was able to inhibit parenchymal nervous tissue infiltration by CD4^+^ and CD8^+^ T-cells as well as by CD11b^+^Ly6C^+^Ly6G^+^ MDCs and that such phenomenon may be dependent on the alteration of the normal inflammatory cell migration route. Notably, when the TcES treatment was withdrawn, white matter was again infiltrated by such cells, suggesting that *T. crassiceps* products aid in the EAE suppression in directing the inflammatory cell migration to the periphery, which makes the constant TcES administration necessary to block the pathology. These findings are according to those recently reported for the mentioned *F. hepatica*-derived peptide-dependent blockade of EAE [29], however, is noteworthy to clarify that in such experiments the use of the *F. hepatica*-derived peptide was a prophylactic assay, whereas in our work TcES was tested as an alternative therapeutic agent.

Collectively, our data indicate that TcES may be a good choice for feature drug development and its mechanisms of action may result in the general interest for anti-MS drug development. A deeper understanding of the phenomenon is necessary to find the most specific and potent molecules from TcES that are associated with this anti-EAE activity.

## Supplementary Material

Figure S1: *Consistency between TcES batches and TcES integrity*. TcES batches obtained in different days and by distinct infected mice produce the same molecular patterns as measured by SDS-PAGE. Figure S2: *Cytokine induction by different TcES doses*. Treatments were administered every other day at the days indicated by the blue arrows (a) and blood sera was extracted on days one (prior to treatment administration), eight and 16. Data shown is representative of two independent experiments with n=6. Statistical significance between groups was determined by two-tailed Student t test, and described by the following criteria ∗∗∗ P ˂ 0.001,∗∗ P ˂ 0.01 and ∗ P ˂ 0.05. Experimental groups were compared with the respective control group (250 µg BSA). Figure S3: *Induction of AAMs by different TcES doses*. The intraperitoneal injection of either 125 or 250 µg of TcES for 16 days, inoculated every other day induce the expression of both MMR and IL-4Rα in F4/80^+^ large cells. Figure S4:*Total numbers of cells per tissue/cavity*. Total cells were extracted from the peritoneal cavity or SNC and plotted (a), whereas total MDSCs (b) and total lymphocytes (c) in those samples were calculated by rule of three parting from event numbers in a 10,000 cell gate (small and non granular for total lymphocytes and big and granular for MDSCs). Data shown is representative of two independent experiments with n=6. Statistical significance between groups was determined by two-tailed Student t test, and described by the following criteria ∗∗∗ P ˂ 0.001,∗∗ P ˂ 0.01 and ∗ P ˂ 0.05.

## Figures and Tables

**Table 1:** TcES foster protection against EAE development.

Treatment	Resolution (%)	Average grade	Maximum grade	CDI	AUC
EAE + BSA	0%	2 ± 0.9	3.5	96	46
EAE + TcES	0%	0.6 ± 0.3	1.5	29.5^∗∗∗^	12.9^∗∗∗^

Resolution = percentage of mice in which disease resolved completely; average grade = average grade of disease of each group. Maximum grade = the peak clinical score for each group. CDI: cumulative disease index (calculated as the sum of all grades for each group); AUC: area under the curve. The data shown are representative of 4 independent experiments. Means and SEM were calculated for groups of *n* = 6 mice. The difference between AUC and CDI were calculated by two-tailed ANOVA test and described by the following criteria: ^∗∗∗^*P* < 0.001. All statistical comparisons were made against the control.

**Table 2:** TcES modulates EAE better than dexamethasone.

Treatment	Resolution (%)	Average grade	Maximum grade	CDI	AUC
EAE + BSA	0%	1.9 ± 1	3.5	71	32.1
EAE + TcES	0%	0.5 ± 0.4	1.5	20.5^∗∗∗^	9.5^∗∗∗^
EAE + Dex	0%	1 ± 0.6	2.5	39^∗∗^	18.2^∗∗∗^

Resolution = percentage of mice in which disease resolved completely; average grade = average grade of disease of each group; maximum grade = the peak clinical score for each group. CDI: cumulative disease index (calculated as the sum of all grades for each group); AUC: area under the curve. The data shown are representative of 2 independent experiments. Means and SEM were calculated for groups of *n* = 6 mice. The difference between AUC and CDI were calculated by two-tailed ANOVA test and described by the following criteria: ^∗∗∗^*P* < 0.001 and ^∗∗^*P* < 0.01. All statistical comparisons were made against the group pinpointed by a blue parenthesis.

**Table 3:** Late TcES administration still arrests EAE development.

Treatment	Resolution (%)	Average grade	Maximum grade	Late CDI	CDI	AUC
EAE + BSA	0%	2 ± 1	3.5	54.5	64	28.5
EAE + TcES	0%	0.6 ± 0.3	1.5	14.5	21.5^∗∗∗^	9.8^∗∗∗^
EAE + TcES-L	0%	1.1 ± 0.5	2	28	30^∗∗∗^	16.8^∗∗^

Resolution = percentage of mice in which disease resolved completely; average grade = average grade of disease of each group; maximum grade = the peak clinical score for each group; late CDI = CDI calculated from treatment onset (day 18) until sacrifice (day 26). CDI: cumulative disease index (calculated as the sum of all grades for each group); AUC: area under the curve. The data shown are representative of 2 independent experiments. Means and SEM were calculated for groups of *n* = 6 mice. The difference between AUC and CDI were calculated by two-tailed ANOVA test and described by the following criteria: ^∗∗∗^*P* < 0.001 and ^∗∗^*P* < 0.01. All statistical comparisons were made against the group pinpointed by a blue parenthesis.

**Table 4:** TcES withdrawal reduces treatment efficacy.

Treatment	Resolution (%)	Average grade	Maximum grade	CDI	Late CDI	AUC
EAE + BSA	0%	2 ± 1.1	3.5	97.5	71.5	29
EAE + TcES	0%	0.8 ± 0.4	1.5	39.5^∗∗∗^	21	12^∗∗∗^
EAE + TcES-W	0%	1.5 ± 0.7	2.5	67^∗∗^	47.5	19.9^∗∗∗^

Resolution = percentage of mice in which disease resolved completely; average grade = average grade of disease of each group; maximum grade = the peak clinical score for each group; late CDI = CDI calculated from treatment withdrawal (day 19) until sacrifice (day 26). CDI: cumulative disease index (calculated as the sum of all grades for each group); AUC: area under the curve. The data shown are representative of 2 independent experiments. Means and SEM were calculated for a group of *n* = 6 mice. The statistical significance between groups was determined by two-way ANOVA test, ^∗∗∗^*P* < 0.001 and ^∗∗^*P* < 0.01. All statistical comparisons were made against the groups pinpointed by the blue parenthesis.
