# Supplementary material for: Helminth Products Potently Modulate Experimental Autoimmune Encephalomyelitis by Downregulating Neuroinflammation and Promoting a Suppressive Microenvironment
Source: Mediators Inflamm. 2017 Jun 28;2017:8494572. doi: 10.1155/2017/8494572 (PMC5506484; doi:10.1155/2017/8494572)
Supplement: Supplementary file 1 — Figure S1: Consistency between TcES batches and TcES integrity. TcES batches obtained in different days and by distinct infected mice produce the same molecular patterns as measured by SDS-PAGE. Figure S2: Cytokine induction by different TcES doses. Treatments were administered every other day at the days indicated by the blue arrows (a) and blood sera was extracted on days one (prior to treatment administration), eight and 16. Data shown is representative of two independent experiments with n=6. Statistical significance between groups was determined by two-tailed Student t test, and described by the following criteria ∗∗∗ P ˂ 0.001,∗∗ P ˂ 0.01 and ∗ P ˂ 0.05. Experimental groups were compared with the respective control group (250 µg BSA). Figure S3: Induction of AAMs by different TcES doses. The intraperitoneal injection of either 125 or 250 µg of TcES for 16 days, inoculated every other day induce the expression of both MMR and IL-4Rα in F4/80+ large cells. Figure S4:Total numbers of cells per tissue/cavity. Total cells were extracted from the peritoneal cavity or SNC and plotted (a), whereas total MDSCs (b) and total lymphocytes (c) in those samples were calculated by rule of three parting from event numbers in a 10,000 cell gate (small and non granular for total lymphocytes and big and granular for MDSCs). Data shown is representative of two independent experiments with n=6. Statistical significance between groups was determined by two-tailed Student t test, and described by the following criteria ∗∗∗ P ˂ 0.001,∗∗ P ˂ 0.01 and ∗ P ˂ 0.05. [file 8494572.f1.docx]

**Supplementary figures**


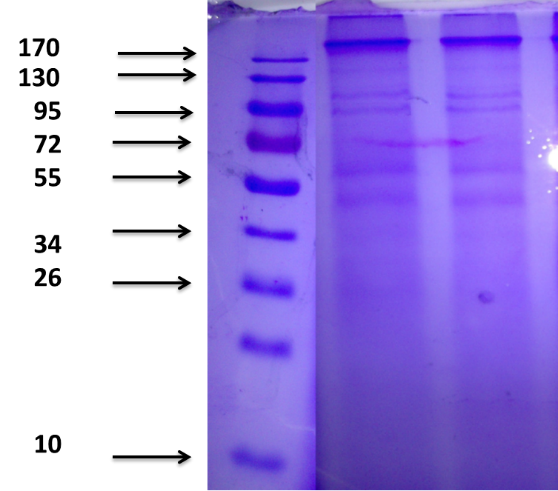


Figure S1: *Consistency between TcES batches and TcES integrity*. TcES batches obtained in different days and by distinct infected mice produce the same molecular patterns as measured by SDS-PAGE.


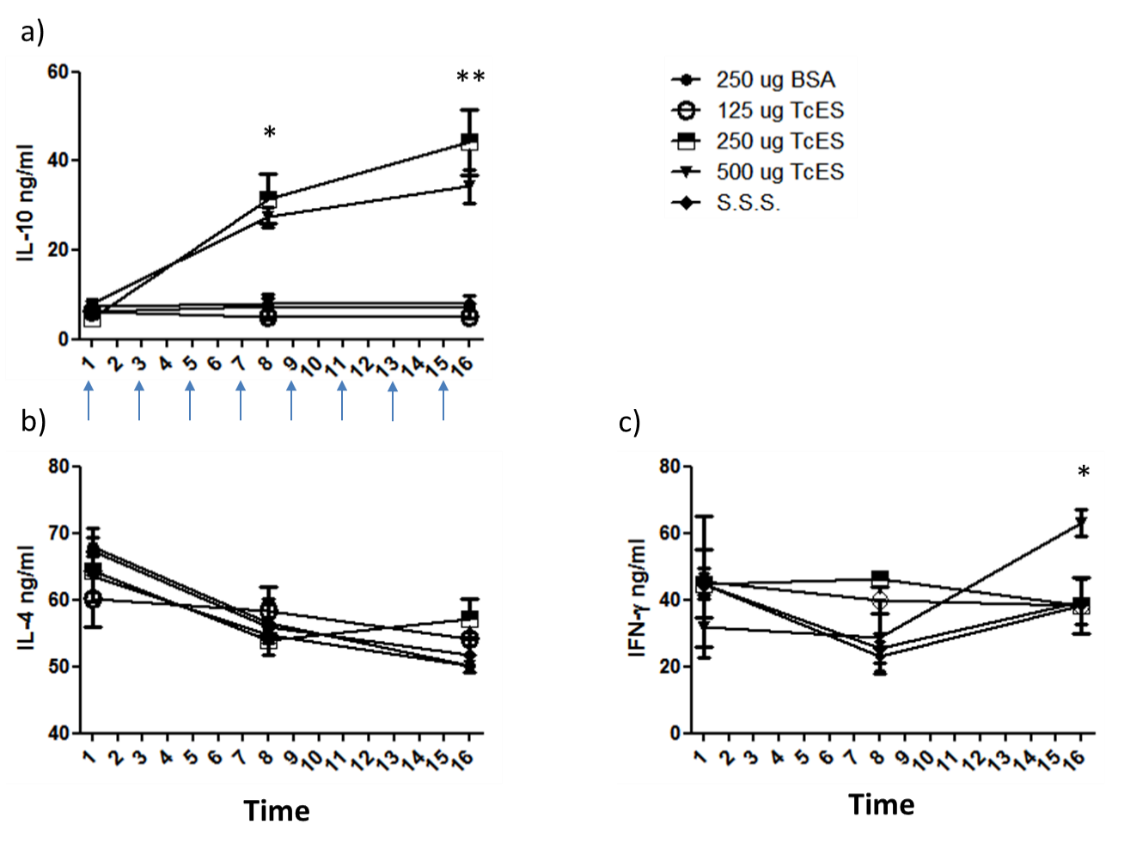


Figure S2: *Cytokine induction by different TcES doses*. Treatments were administered every other day at the days indicated by the blue arrows (a) and blood sera was extracted on days one (prior to treatment administration), eight and 16. Data shown is representative of two independent experiments with n=6. Statistical significance between groups was determined by two-tailed Student t test, and described by the following criteria *** P ˂ 0.001,** P ˂ 0.01 and * P ˂ 0.05. Experimental groups were compared with the respective control group (250 μg BSA).


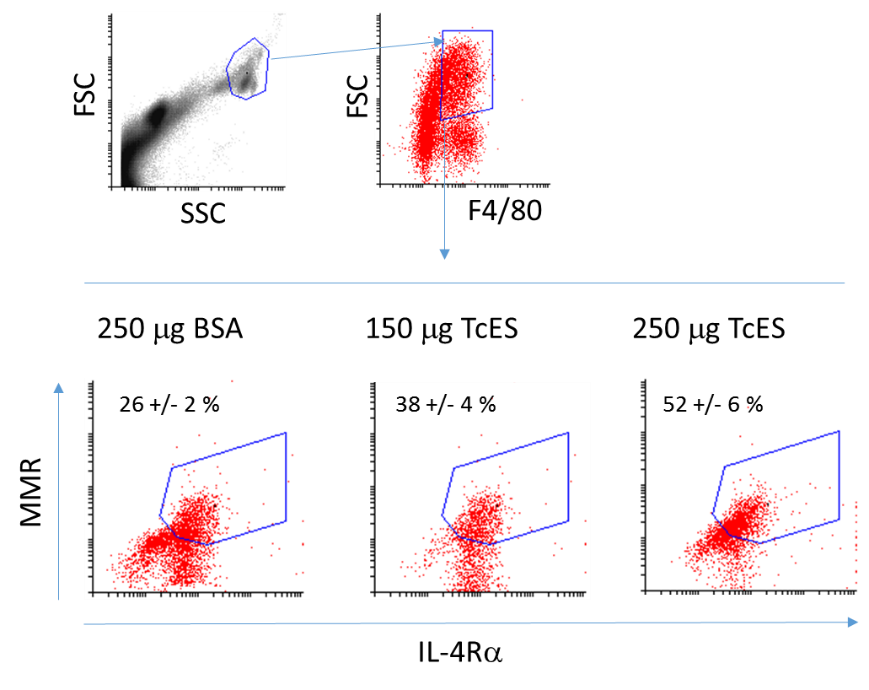


Figure S3: *Induction of AAMs by different TcES doses*. The intraperitoneal injection of either 125 or 250 μg of TcES for 16 days, inoculated every other day induce the expression of both MMR and IL-4Rα in F4/80^+^ large cells.


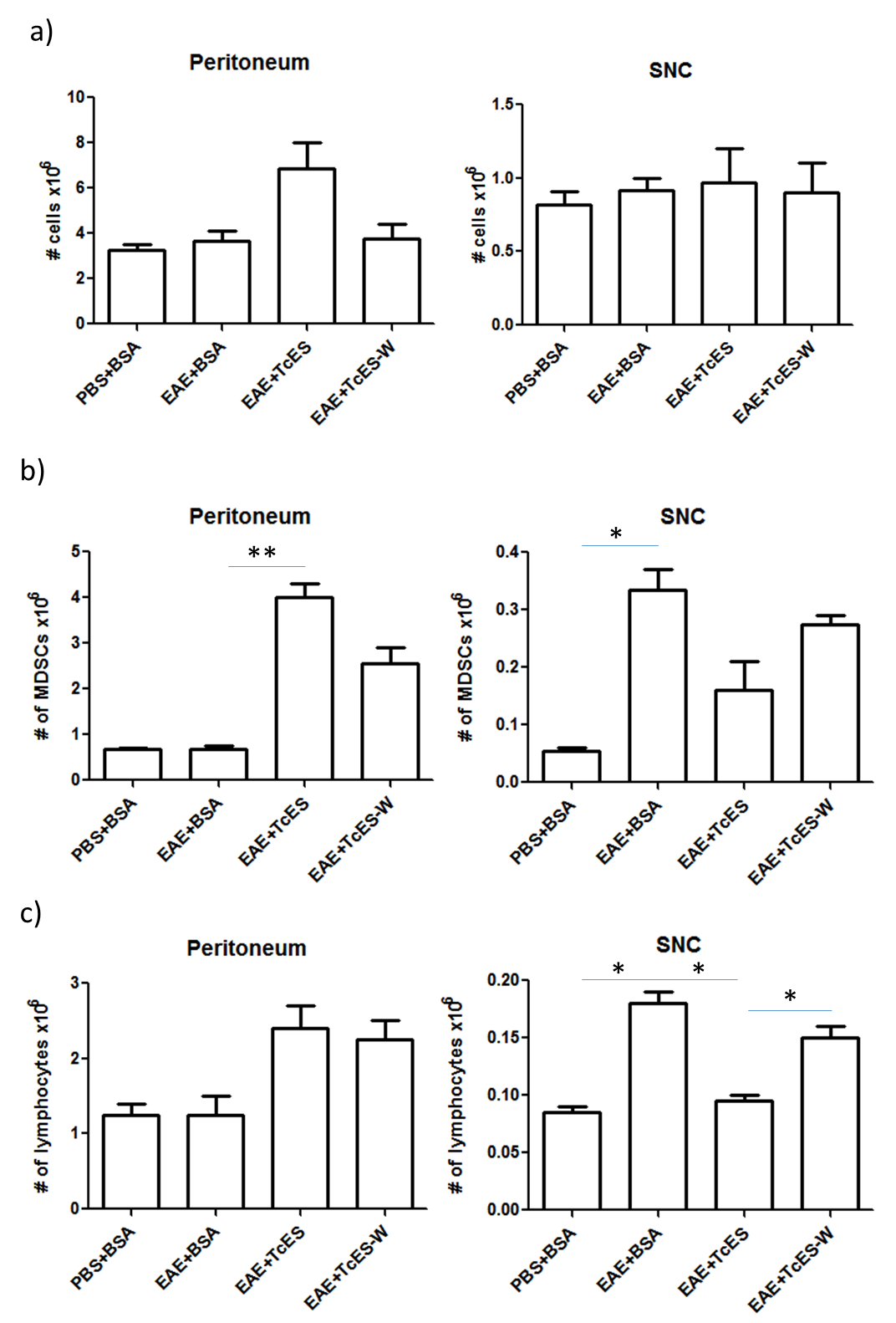


Figure S4:T*otal numbers of cells per tissue/cavity*. Total cells were extracted from the peritoneal cavity or SNC and plotted (a), whereas total MDSCs (b) and total lymphocytes (c) in those samples were calculated by rule of three parting from event numbers in a 10,000 cell gate (small and non granular for total lymphocytes and big and granular for MDSCs). Data shown is representative of two independent experiments with n=6. Statistical significance between groups was determined by two-tailed Student t test, and described by the following criteria *** P ˂ 0.001,** P ˂ 0.01 and * P ˂ 0.05.
